# Proximale osteosynthetisch versorgte Femurfrakturen: Der Versorgungszeitpunkt verzögert sich bei vorbestehender Antikoagulation: Ergebnisse der Daten der externen stationären Qualitätssicherung aus Nordrhein-Westfalen mit einer Fallzahl von 24.786 Fällen im Rahmen sekundärer Datennutzung

**DOI:** 10.1007/s00113-020-00923-2

**Published:** 2020-11-27

**Authors:** J. G. Korbmacher, U. Schulze-Raestrup, H. Nowak, R. Smektala

**Affiliations:** 1 Klinik für Unfallchirurgie und Orthopädie, Knappschaftskrankenhaus Bochum-Langendreer, Universitätsklinik der Ruhr Universität Bochum, In der Schornau 23–25, 44892 Bochum, Deutschland; 2 Qualitätssicherung NRW, Ärztekammer Westfalen-Lippe, Münster, Deutschland; 3 Klinik für Anästhesiologie, Knappschaftskrankenhaus Bochum-Langendreer, Universitätsklinik der Ruhr-Universität Bochum, In der Schornau 23-25, 44892 Bochum, Deutschland

**Keywords:** Gerinnungsmanagement, IQTIG, Qualitätsmanagement, Pertrochantäre Femurfraktur, Registerdaten, Coagulation management, Guidelines, Quality management, Pertrochanteric femoral fracture, Register data

## Abstract

**Hintergrund und Fragestellung:**

Proximale Femurfrakturen stellen mit ca. 100.000 Betroffenen/Jahr in Deutschland ein häufiges Krankheitsbild dar. Durch eine zeitnahe Versorgung (<24 h) konnte die Mortalität erheblich gesenkt werden. Ziele der Arbeit waren, die Prävalenz der Antikoagulation und hiermit assoziierte Komplikationen bei osteosynthetisch versorgter, proximaler Femurfraktur und deren Impact auf die präoperative Verweildauer zu analysieren und Potenziale zum optimalen perioperativen Gerinnungsmanagements aufzuzeigen.

**Material und Methoden:**

Die Daten der externen vergleichenden Qualitätssicherung Nordrhein-Westfalen für die Jahre 2015 und 2016 wurden ausgewertet. Dabei wurden ausschließlich Fälle analysiert, bei denen eine hüftgelenknahe Femurfraktur osteosynthetisch versorgt wurde. Insgesamt wurden 24.786 Fälle hüftgelenknaher Femurfrakturen in die Studie eingeschlossen.

**Ergebnisse:**

Von den Patienten mit einer antithrombotischen Dauertherapie (ATDT) wurden in der größten Subgruppe mit ASS-Medikation (*n* = 4005) 17 %, in der zweitgrößten Gruppe mit Vitamin-K-Antagonisten-Einnahme (*n* = 2157) 44,6 % und in der drittgrößten Gruppe mit Einnahme von direkten oralen Antikoagulanzien (DOAKs, *n* = 994) 18,2 % verzögert operiert.

**Schlussfolgerungen:**

Das größte Potenzial zur Verkürzung der präoperativen Verweildauer ergibt sich in der Gruppe der Patienten, die ASS (17 % auffällig) oder einen Vitamin-K-Antagonisten (VKA, 44,6 % auffällig) einnehmen. Eine Antagonisierung der Wirkung von VKA lässt sich innerhalb kurzer Zeit durch die Gabe von Prothrombinkomplex (PPSB) erreichen. Auch unter der Einnahme von DOAKs muss das noch gängige Prozedere einer verzögerten operativen Versorgung kritisch hinterfragt werden. Die Etablierung eines Gerinnungsmanagements ist zu fordern. Neben der medizinischen Intervention (Gabe von Antidota) müssen Strukturen geschaffen werden, die eine zeitnahe Versorgung ermöglichen.

## Abstract (en)

**Background and objective:**

Proximal femoral fractures are common in Germany with approximately 100,000 affected patients per year. The mortality could be considerably reduced by timely treatment (<24 h). The objectives of this work were to demonstrate the prevalence of anticoagulation and associated complications in osteosynthetically treated proximal femoral fractures, the impact of anticoagulation on the preoperative period and potential optimization of perioperative anticoagulation management.

**Material and methods:**

External quality control data for North Rhine-Westphalia for the years 2015–2016 were evaluated. Only cases in which a femoral fracture near the hip joint was treated osteosynthetically were analyzed. A total of 24,786 cases of femoral fractures near the hip joint were included in the study.

**Results:**

In the largest subgroup with acetylsalicylic acid (ASS) medication (*n* = 4005) 17% underwent delayed surgery, in the second largest group with vitamin K antagonists (VKA, *n* = 2157) 44.6% underwent delayed surgery and in the third largest group with direct oral anticoagulant (DOACs) medication (*n* = 994) 18.2% underwent delayed surgery.

**Conclusion:**

The biggest potential of shortening the preoperative period can be found in the ASS and vitamin K antagonist subgroups (17% and 44.6% delayed surgery, respectively). The antagonization of the effect of VKA can be achieved within a short time by the administration of prothrombin complex (PPSB). Even when taking DOACs, the current common procedure of delayed surgical treatment must be critically questioned. A coagulation management should be established in the SOP. In addition to medical interventions (administration of antidotes), structures must be created that enable prompt care.

## Hintergrund und Fragestellung

Proximale Femurfrakturen stellen im Leben älterer Betroffener einen erheblichen Einschnitt dar, beeinträchtigen die Lebensqualität und haben weitreichende gesundheitliche und soziale Folgen. Jährlich erleiden ca. 100.000 Menschen in Deutschland eine proximale Femurfraktur [1].

Es konnte gezeigt werden, dass die Letalität mit längerer präoperativer Verweildauer steigt [20]. Die Letalität war um 6 % niedriger bei Patienten, die innerhalb von 24 h operiert wurden, im Vergleich zu Patienten, die später operiert wurden [20]. Daher wurde bereits 2014 jeweils eine S2e-Leitlinie zur Schenkelhalsfraktur und zur pertrochantären Oberschenkelfraktur von der Deutschen Gesellschaft für Unfallchirurgie (DGU) in Zusammenarbeit mit der Österreichischen Gesellschaft für Unfallchirurgie (ÖGU) erstellt, die festlegt, dass Patienten mit einer Schenkelhalsfraktur bzw. pertrochantären Femurfraktur „so schnell wie möglich innerhalb von 24 h operiert werden sollten, wenn der Allgemeinzustand des Patienten dies zulässt“ [2, 8].

Der Qualitätsbericht des IQTIG für das Jahr 2018 wies auf 7 Indikationen hin, bei denen Handlungsbedarf bestand, da die vorgegebenen Qualitätsziele wiederholt nicht erreicht wurden. Eine dieser Indikationen ist die präoperative Verweildauer bei Vorliegen einer proximalen Femurfraktur, die osteosynthetisch versorgt werden soll [15]. Die Gründe für die Einführung dieses Indikators wurde durch das IQTIG in der Rationalen wissenschaftlich begründet [14]. Um die beobachteten Qualitätsmängel abzustellen, veröffentlichte der Gemeinsame Bundesausschuss am 22.11.2019 zur Frage der Versorgung proximaler Femurfrakturen eine Richtlinie, die zum 01.07.2020 in Kraft treten sollte.

Diese Richtlinie umfasst Maßnahmen zur Qualitätssicherung zur Versorgung von Patienten mit einer hüftgelenknahen Femurfraktur. Erklärtes Ziel dieser Richtlinie ist die „Gewährleistung einer qualitativ hochwertigen und frühestmöglichen operativen Versorgung von Patienten mit einer hüftgelenknahen Femurfraktur, in Regel innerhalb von 24 h […]“ [10].

Das Qualitätsziel wurde in NRW mit 19,96 % (KI 19,26–20,68 %) im Jahr 2015 und 18,95 % (KI 18,27–19,65 %) im Jahr 2016 verfehlt. In absoluten Zahlen bedeutet dies, dass im Jahr 2015 in 2461 Fällen (von 12.329) und im Jahr 2016 in 2361 Fällen das Qualitätsziel nicht erreicht werden konnte.

Ziel der vorgelegten Auswertung auf Basis der Daten der externen Qualitätssicherung war es, den Einfluss einer antithrombotischen Dauertherapie (ATDT) auf die präoperative Verweildauer aufzuzeigen, da es sich im strukturierten Dialog gezeigt hatte, dass ein häufiger Grund für eine Verzögerung der Operation eine vorbestehende Medikation mit gerinnungsaktiven Medikamenten war.

Folgende Fragen sollen beantwortet werden:

1. Wie viele Patienten nehmen eine ATDT ein? Welche gerinnungshemmenden Medikamente werden eingenommen?
2. Verlängert sich durch die Einnahme einer ATDT die präoperative Verweildauer?
3. Treten mehr Komplikationen aufgrund der Einnahme von Antikoagulanzien auf?

## Material und Methoden

### Datengrundlage und Erhebungsinstrumente

Die Auswertung beruht auf den Daten der externen vergleichenden Qualitätssicherung Nordrhein-Westfalen für die Jahre 2015 und 2016. Bis einschließlich 2014 wurden osteosynthetische und endoprothetische Prozeduren nach einem Oberschenkelhalsbruch gemeinsam im QS-Verfahren „Hüftgelenknahe Femurfraktur“ erfasst. Ab 2015 richtete sich die Auswertung des Instituts für Qualitätssicherung und Transparenz im Gesundheitswesen (IQTIG) nach der Art der Versorgung. Somit werden seit 2015 jeweils die osteosynthetischen Verfahren (DHS, PFN etc.) und die endoprothetischen Verfahren (Duokopfprothese, HTEP etc.) getrennt voneinander registriert. Das IQTIG wurde durch den gemeinsamen Bundesausschuss (G-BA) mit dieser Aufgabe betraut. Das IQTIG erfasst bundesweite Daten, jedoch sind nach wie vor regionale Auswertungen über die Geschäftsstellen, die in Nordrhein-Westfalen bei den Landesärztekammern angesiedelt sind, möglich.

Insgesamt wurden 24.786 Fälle hüftgelenknaher Femurfrakturen, die osteosynthetisch versorgt wurden, in die Auswertung eingeschlossen. Die Verteilung auf die Jahre 2015 und 2016 ist annähernd gleich (2015: 12.329 und 2016:12.457). Patienten unter 20 Jahren wurden infolge der Rechenregeln des IQTIG nicht erfasst und somit aus der Auswertung ausgeschlossen. Die Auswertung der Daten beider Jahrgänge (2015 + 2016) wurde auf Basis der Spezifikation von 2016 erstellt.

Der Toleranzbereich für die präoperative Verweildauer wurde durch das IQTIG (vormals Aqua-Institut) mit 15 % festgelegt, d. h., für maximal 15 % der Patienten können medizinische Gründe vorliegen, die zu einer Verzögerung der Versorgung führen. Als verzögert operiert gelten dabei Patienten, die später als 24 h nach Aufnahme oder Frakturereignis während des stationären Aufenthaltes operiert werden. Für die Patientengruppen der direkten Thrombininhibitoren und ATDT in der Kategorie Sonstige (z. B. Rivaroxaban, Fondaparinux) wurde durch das IQTIG eine präoperative Verweildauer von 48 h als Toleranzbereich festgelegt und somit in der vorliegenden Auswertung auch entsprechend berücksichtigt.

### Auswertung und grafische Darstellung

Die Daten wurden mit SPSS 23 ausgewertet. Hierbei kam neben den Verfahren der deskriptiven Statistik für die analytische Auswertung der unterschiedlichen Einflussfaktoren die binäre logistische Regression zur Anwendung. Als abhängige Variablen wurden die Variablen „Tod“, „allgemeine Komplikationen“^1^ und hier eine zusammengefasste Subgruppe der Items für kardiovaskuläre Komplikationen, Lungenembolie und Thrombose ausgewertet. Des Weiteren wurden die „spezifischen Komplikationen“^2^ untersucht, wobei ein besonderes Augenmerk auf das Item „Hämatom/Blutung“ gelegt wurde. Als unabhängige Variablen wurden Alter, Geschlecht, ASA-Klassifikation, Frakturlokalisation, Fachabteilung, präoperative Verweildauer und ATDT (Art der Medikation) definiert.

Um einen Überblick zur diagnostischen Güte zu erhalten, wurden jeweils „Receiver-operating-characteristics“(ROC)-Kurven generiert. Als Maßzahl zur Beschreibung einer Kurve hat sich die Fläche unter der Kurve (engl. „area under the curve“, ROC AUC) durchgesetzt. Logistische Regressionsmodelle sind umso besser, je näher ihre ROC AUC bei 1 liegt.

Für das Regressionsmodell „Tod“ beträgt der Wert 0,789 und für die Subgruppe der kardiovaskulären und thrombotischen Komplikationen und Lungenembolie 0,748. Somit können diese Ergebnisse als gut bewertet werden. Hingegen liegen die Ergebnisse der anderen Modelle unter 0,70, womit die Ergebnisse als schwach bewertet werden müssen.

Da auf Basis des vorliegenden Datensatz mehrere Auswertungen durchgeführt werden, ist von einer multiplen Testsituation auszugehen. Aus diesem Grund ist mit einer α‑Fehler-Kumulierung zu rechnen. Um dies zu vermeiden, kommt die Bonferroni-Korrektur zur Anwendung. Sie ist die einfachste und konservativste Form, das multiple α‑Niveau anzupassen. Dabei wird das globale α‑Niveau zu gleichen Teilen auf die Einzeltests verteilt. Bei einem angestrebten α‑Fehler von 0,05 bedeutet dies für diese Untersuchung, dass bei 5 Tests ein korrigierter Wert von 0,01 in die Auswertung eingehen muss. Dies wurde in der Form umgesetzt, dass das Konfidenzintervall bei der binären, logistischen Regression auf 99,0 % festgelegt wurde.

Die Grafiken wurden mit Excel 2010 und SPSS 23 generiert.

## Ergebnisse

### Beschreibung der Grundgesamtheit

**Figure 1:**
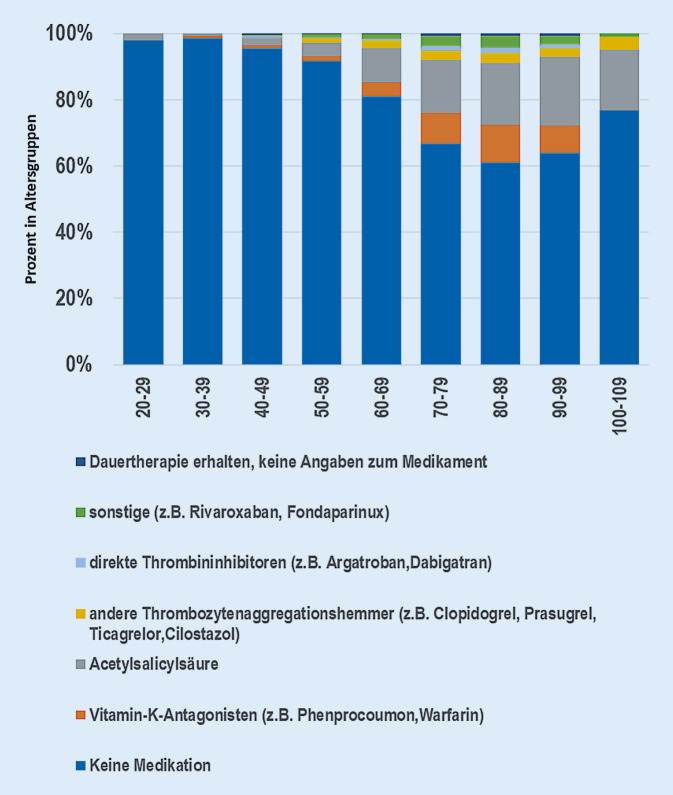

Das Durchschnittsalter der Patienten lag bei 79,3 Jahren mit einer Standardabweichung von 12,4 Jahren. Die prozentuale Verteilung innerhalb der Altersgruppen zeigt Abb. 1. Das Geschlechterverhältnis betrug männlich zu weiblich 32,3 % zu 67,7 %.

### Auswertungen nach Fragestellungen

#### 1. Wie viele Patienten nehmen gerinnungshemmende Medikamente ein? Welche gerinnungshemmenden Medikamente werden eingenommen?

**Figure 2:**
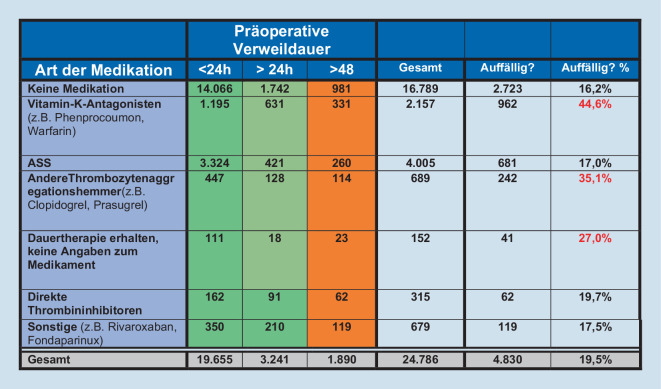

In NRW wurden in den Jahren 2015 und 2016 insgesamt 24.786 Patienten mit hüftgelenknaher Femurfraktur operativ versorgt, von denen 16.789 keine ATDT erhielten. 32,3 % der Patienten (7997 Pat.) mit hüftgelenknaher Femurfraktur erhielten eine ATDT. Andere Thrombozytenaggregationshemmer (689 Patienten) und „Sonstige“ (679 Pat.) lagen im Mittelfeld, wohingegen die direkten Thrombininhibitoren mit 315 Patienten die kleinste Gruppe darstellten. Bei 152 Patienten wurde eine Dauerbehandlung angegeben, jedoch die Substanzgruppe nicht beschrieben. Vitamin-K-Antagonisten (2157 Pat.) und ASS (4005 Pat.) wurden am häufigsten eingenommen. Von den Patienten, die eine ATDT einnehmen, wurden insgesamt 26,3 % der Fälle (2107 Pat.) verzögert operiert. Einen Überblick über die eingenommene Art der Antikoagulation und die präoperative Verweildauer gibt Abb. 2.

#### 2. Verlängert sich durch die Einnahme einer antithrombotischen Dauertherapie die präoperative Verweildauer?

Patienten, die einen Vitamin-K-Antagonisten (Phenprocoumon oder Warfarin) einnahmen, wurden in 962 Fällen (44,6 %) außerhalb der vorgesehenen Zeit operiert. Der prozentuale Anteil der Patienten, die einen „anderen Thrombozytenaggregationshemmer“ (z. B. Clopidogrel, Prasugrel, Ticagrelor oder Cilostazol) erhielten und nicht innerhalb von 24 h operiert wurden, lag zwar bei 35,1 %, aber bei einer Gesamtzahl von 689 Patienten ist dieses Ergebnis sekundär. Das Gleiche galt für die Patienten mit einer Dauertherapie ohne Angaben zur Medikation (Abb. 2).

#### 3. Treten mehr Komplikationen aufgrund der Einnahme von Antikoagulanzien auf?

##### Letalität.

**Figure 3:**
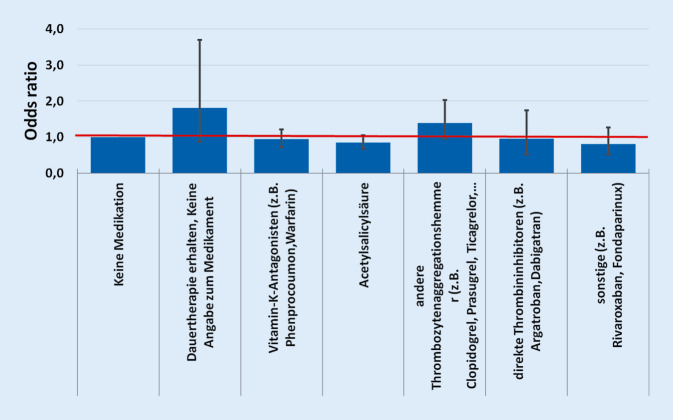

Bei der Berücksichtigung der antithrombotischen Substanzgruppen zeigte sich, dass die „odds ratio“ bei den Patienten, die mit einem „anderen Thrombozytenaggregationshemmer“ behandelt wurden, bei 1,393 liegt. Bei den Patienten, die zwar eine Dauertherapie erhalten haben jedoch keine Angaben zum Medikament gemacht wurden, liegt dieser Wert bei 1,805. Bei den anderen Substanzen liegt der Wert unter 1, was für einen positiven Effekt sprechen könnte. Bei dem nach Bonferroni korrigierten Bereich für das Konfidenzintervall von 99 % ist jedoch keines der Ergebnisse signifikant (Abb. 3).

Die Werte des Hosmer-Lemeshow-Tests (*p* = 0,869) und der Fläche unter der Kurve/ROC (0,789) bestätigen, dass die Güte des Regressionsmodells als gut einzuordnen ist.

##### Allgemeine Komplikationen.

Werden die antithrombotischen Medikamente im Zusammenhang mit allgemeinen postoperativen Komplikationen gebracht, so zeigt sich ein Effekt bei den folgenden Substanzen. Bei Vitamin-K-Antagonisten lag die Odds ratio bei 1,229 (99 %-KI: 1,030 und 1,466, *p* = 0,003), bei ASS bei 1,168 (99 %-KI: 1,013 und 1,348; *p* = 0,005), bei anderen Thrombozytenaggregationshemmern bei 1,661 (99 %-KI: 1,270 und 2,171; *p* = 0,000).

**Figure 4:**
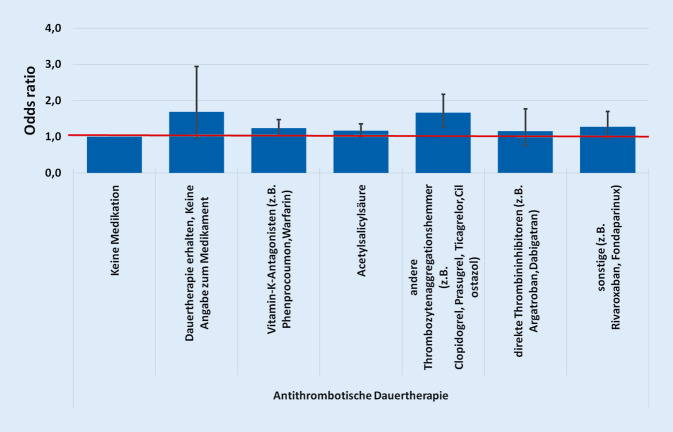

Bei den direkten Thrombininhibitoren liegt der Wert bei 1,157 (99 %-KI: 0,755 und 1,773; *p* = 0,378), bei sonstiger ATDT bei 1,270 (99 %-KI: 0,951 und 1,696; *p* = 0,034) und bei „Dauertherapie erhalten, keine Angaben zum Medikament“ bei 1,682 (99 %-KI: 0,964 und 2,936; *p* = 0,016), womit diese Ergebnisse nicht signifikant sind (Abb. 4).

Die Werte des Hosmer-Lemeshow-Tests (*p* = 0,014) und der Fläche unter der Kurve/ROC (*p* = 0,693) zeigen, dass die Güte des Regressionsmodells als schwach einzuordnen ist.

##### Kardiovaskuläre Komplikationen/Thrombose/Lungenembolie.

Insgesamt konnten in dem untersuchten Datensatz 911 kardiovaskuläre Komplikationen (3,7 %), 30 thrombotische Komplikationen (0,1 %) und 66 Lungenembolien (0,3 %) beobachtet werden. Diese 3 Gruppen wurden zusammengefasst, wobei Mehrfachnennungen von Komplikationen bei einem Patienten als ein Ereignis gewertet wurden. Insgesamt konnte bei 986 Patienten (4,0 %) wenigstens eine dieser Komplikationen beschrieben werden.

**Figure 5:**
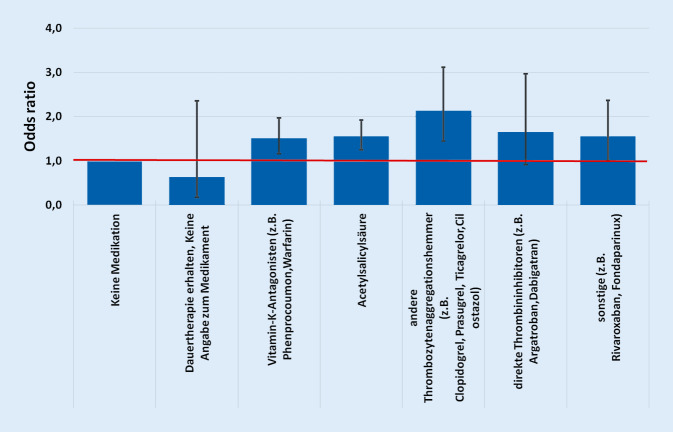

Bei allen Patienten – außer bei denjenigen, bei denen keine Spezifizierung der ATDT angegeben wurde – stieg das Risiko für diese Komplikationen an. Die Odds ratio lag zwischen 1,506 und 2,224. Für die direkten Thrombininhibitoren konnte jedoch kein signifikantes Ergebnis beschrieben werden. Hervorzuheben ist, dass in der Gruppe „andere Thrombozytenaggregationshemmer“ das Risiko für ein solches Ereignis ansteigt (OR: 2,124; *p* = 0,000). Lediglich bei den Patienten, bei denen keine Spezifizierung der ATDT angegeben wurde, konnte eine Odds ratio kleiner 1,000 beschrieben werden, wobei die Werte des 99 %-KI bei 0,166 und 2,348 lagen, was auf die kleine Fallzahl zurückzuführen ist (Abb. 5).

Die Werte des Hosmer-Lemeshow-Tests (Sig: 0,438) und der Fläche unter der Kurve/ROC (0,748) bestätigen, dass die Güte des Regressionsmodells als gut einzuordnen ist.

##### Spezifische Komplikationen.

Die Anzahl der Patienten, bei denen eine spezifische postoperative Komplikation auftrat, betrug 550.

Die Werte für die Odds ratio betragen für die Patienten mit einer „Dauertherapie erhalten, keine Angabe zum Medikament“ OR = 1,636, für „Vitamin-K-Antagonisten“ OR = 1,261 und für „andere Thrombozytenaggregationshemmer“ OR = 1,109. Die Ergebnisse für die anderen Substanzen liegen unter 1: Acetylsalicylsäure OR = 0,956, direkte Thrombininhibitoren OR = 0,739 und „Sonstige“ OR = 0,671. Keines dieser Ergebnisse ist signifikant.

Die Werte des Hosmer-Lemeshow-Tests (*p* = 0,212) und der Fläche unter der Kurve/ROC (0,646) zeigen, dass die Güte des Regressionsmodells als schwach einzuordnen ist.

##### Hämatom/Nachblutung.

Insgesamt wurde bei 233 Patienten (von gesamt 24.786 Pat.) eine Nachblutung beobachtet, entsprechend einem Anteil von 0,94 %. 130 der 233 Patienten erhielten keine ATDT, die übrigen 103 Patienten eine entsprechende Gerinnungsprophylaxe. Von den Patienten, die eine ATDT erhalten hatten, fanden sich bei Vitamin-K-Antagonisten 38 Patienten (gesamt 2157 Pat.), bei Acetylsalicylsäure 42 Patienten (gesamt 4005 Pat.), bei anderen Thrombozytenaggregationshemmern 12 Patienten (gesamt 677 Pat.), bei direkten Thrombininhibitoren 2 Patienten (gesamt 315 Pat.), bei „Sonstigen“ 6 Patienten (gesamt 673 Pat.) und bei „Dauertherapie erhalten, keine Angaben zum Medikament“ 3 Patienten (gesamt 152 Pat.) mit einem Hämatom oder einer Nachblutung.

**Figure 6:**
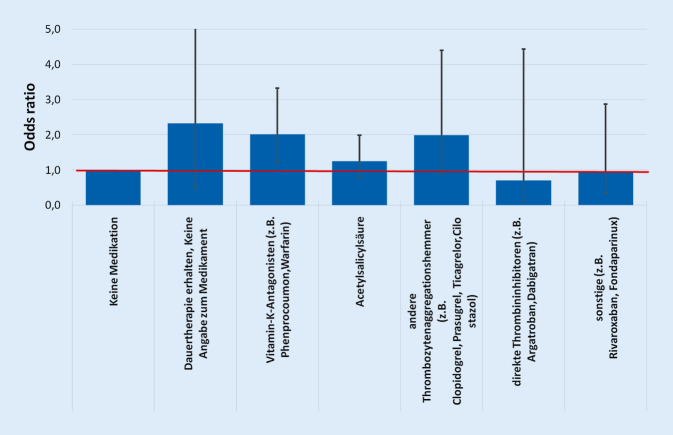

Die Gabe von Vitamin-K-Antagonisten zeigte ein signifikantes Ergebnis im Vergleich mit den Patienten, die keine Dauertherapie erhalten hatten. Dabei ist die Odds ratio bei der Therapie mit Vitamin-K-Antagonisten doppelt so hoch (OR: 2,010), wobei das 99 %-KI zwischen 1,213 und 3,329 lag. Bei einer Therapie mit anderen Thrombozytenaggregationshemmern war das Ergebnis ebenfalls nahezu verdoppelt (OR: 1,985), jedoch lag das Minimum des 99 %-KI bei OR = 0,895, womit keine Signifikanz vorlag. Auch bei den weiteren Substanzen konnte keine Signifikanz beschrieben werden. Insgesamt ist die Fallzahl der Hämatome und Nachblutungen sehr gering, sodass die Ergebnisse kritisch zu werten sind (Abb. 6).

Die Werte des Hosmer-Lemeshow-Tests (Sig: 0,212) und der Fläche unter der Kurve/ROC (0,646) zeigen, dass die Güte des Regressionsmodells als schwach einzuordnen ist.

## Diskussion

Die Versorgungsforschung ist der „Motor für ein lernendes und sich stetig entwickelndes Gesundheitssystem“ [3]. Die vorliegende Auswertung stellt mit der Analyse von 24.786 Fällen proximaler Femurfrakturen in NRW einen wichtigen Beitrag zur Versorgungsforschung dar.

Die Auswertung zeigt, dass für den Versorgungszeitraum 2015/2016 19,5 % der Patienten nicht zeitgerecht operiert wurden. Dabei liegt ein erheblicher Unterschied zwischen den einzelnen Medikationen einer ATDT vor. Wird der Indikator vor 2016 hinzugezogen, nach dem eine Operation innerhalb von 48 h durchzuführen wäre, so wäre nur ein Anteil von 7,6 % der Patienten auffällig. Daraus lässt sich schließen, dass nach der Trennung der osteosynthetisch und endoprothetischen Versorgung der hüftgelenknahen Frakturen die Änderung in der Spezifikation und die damit verbundenen Bedingungen in einigen Kliniken nicht berücksichtigt werden. Insgesamt werden in dem Zeitfenster zwischen 24 und 48 h 3241 Patienten operiert. Davon wurde ein Anteil von 90,7 % (2940 Patienten) nach der neuen Spezifikation nicht zeitgerecht operiert (nach der alten Spezifikation wären diese Patienten nicht auffällig gewesen).

Nach der neuen Spezifikation sind die Patienten, bis auf wenige Ausnahmen, innerhalb von 24 h zu operieren. Eine ATDT rechtfertigt nur im Fall einer Therapie mit direkten Thrombininhibitoren bzw. Sonstigen (z. B. Rivaroxaban, Fondaparinux) eine Ausweitung des Therapiefensters bis 48 h. Für diese Patienten sind die Komplikationsereignisse geringfügig erhöht (19,7 % bzw. 17,5 %).

Vor allem Bedenken hinsichtlich möglicher intraoperativer, aber auch postoperativer Blutungen spielen eine wichtige Rolle [16]. Dabei liegt der Fokus v. a. auf Vitamin-K-Antagonisten (VKA) und den direkten oralen Antikoagulanzien (DOAKs). Als zentraler Befund der vorgelegten Auswertung kristallisiert sich heraus, dass ein Großteil der Patienten, die verzögert operiert werden, einen VKA einnehmen. In dieser Gruppe zeigte sich in der vorgelegten Auswertung ein Anteil der Patienten, die verzögert operativ versorgt worden sind, von 44,6 %. Diese Ergebnisse sind vergleichbar mit den Ergebnissen anderer Observationsstudien, obgleich die Daten nicht immer komplett vergleichbar sind, da in unterschiedlichem Kontext teilweise erst ab 48 h von einer verzögerten Operation gesprochen wird [5, 9, 16].

Die in dieser Auswertung ermittelten Daten stellen die Berechtigung einer Behandlungsverzögerung aufgrund befürchteter Blutungskomplikationen infrage. Eine Reversierung der Wirkung von VKA lässt sich problemlos innerhalb kurzer Zeit unmittelbar präoperativ durch die Gabe von Prothrombinkomplex erreichen – zahlreiche Arbeiten bei Patienten mit Eingriffen am proximalen Femur konnten zeigen, dass eine erfolgreiche Aufhebung der VKA-Wirkung durch diese Substanz erzielt werden kann [21]. Einzelne dieser Studien berichten über eine erhöhte Rate an kardialen Ereignissen bei den mit Prothrombinkomplex behandelten Patienten, allerdings sollte dies in den meisten Fällen weniger auf die Reversierung an sich zurückzuführen sein als auf die Tatsache, dass Patienten unter einer VKA-Therapie implizit insgesamt wegen der zugrunde liegenden Erkrankung ein erhöhtes Risiko für entsprechende Ereignisse aufweisen. PPSB wirkt innerhalb von 30 min, und die Wirkung hält mindestens 6 h an. Die gleichzeitige Gabe von Vitamin K erlaubt eine stabile Antagonisierung ohne Rebound-Effekt [22]. Es gilt zu beachten, dass Warfarin eine deutlich kürzere Halbwertszeit als Phenprocoumon ausweist und damit die Vergleichbarkeit einschränkt.

Auch unter der Einnahme von DOAKs muss das noch gängige Prozedere einer verzögerten operativen Versorgung von proximalen Femurfrakturen kritisch hinterfragt werden. DOAKs werden inzwischen in Deutschland häufiger eingenommen als VKA [24]. Diese Medikamente zeichnen sich durch eine im Vergleich zu VKA kurze Halbwertszeit von nur 5–17 h aus (je nach Substanz und Nierenfunktion des Patienten); sie sind somit vergleichbar zu den Halbwertszeiten von niedermolekularen Heparinen [26]. Gerade unter Berücksichtigung des individuellen letzten Einnahmezeitpunktes eines DOAK’s ließe sich somit in den allermeisten Fällen eine Operation innerhalb von 24 h in dieser Patientengruppe gewährleisten, z. B. indem man den Zeitpunkt der Operationen eher in die zweite Hälfte des 24-stündigen Zeitraums legt. Zusätzlich existiert im Falle eines schweren, lebensbedrohlichen Blutungsnotfalls neben der Möglichkeit der Gabe von Prothrombinkomplex auch eine spezifische Antagonisierung: im Fall von Dabigatran ist dies Idarucizumab; bei den Faktor-Xa-Inhibitoren steht Andexanet alfa in Aussicht, dessen Zulassung auf dem europäischen Markt im März 2019 von der Europäischen Arzneimittel-Agentur (EMA) empfohlen worden ist und somit in absehbarer Zukunft auch verfügbar sein sollte [6, 7, 23]. In der vorgelegten Auswertung wurden 48 % der Patienten, die direkte Thrombininhibitoren eingenommen haben, verzögert (>24 h) operiert. Dabei wurde nicht differenziert, welcher direkte Thrombininhibitor eingenommen wurde.

Wenn keine genauen Angaben zum Medikament gemacht werden konnten, aber eine ATDT verabreicht wurde, stiegen in den zur Verfügung stehenden Daten sowohl die Wahrscheinlichkeiten, zu versterben als auch postoperativ allgemeine Komplikationen zu erleiden, an. Hieraus könnte eine Empfehlung zur labortechnischen Substanzbestimmung abgeleitet werden, wobei hier die Fallzahl mit *n* = 679 Fällen sehr gering war.

Für die Gruppen der direkten (Apixaban, Edoxaban, Rivaroxaban) und indirekten Faktor-Xa-Inhibitoren (Fondaparinux) sowie niedermolekularen Heparine ist Andexanet alfa ein vielversprechendes Antidot. Andexanet ist ein modifizierter Faktor Xa und bindet somit die Faktor-Xa-Inhibitoren, hat aber keine enzymatische Aktivität [19]. Andexanet reduziert die Anti-Faktor-Xa-Aktivität innerhalb von 2–5 min [25]. Die Halbwertszeit ist mit 1 h sehr kurz [25]. In einer noch laufenden Phase-IIIb/IV-Studie („ANNEXA-Studie“) zeigten Zwischenergebnisse die signifikante Senkung der Anti-Faktor-Xa-Aktivität bei Patienten mit lebensbedrohlichen Blutungen [11–13]. In der vorliegenden Auswertung fielen unter „Sonstige“ Patienten mit Einnahme von z. B. Rivaroxaban und Fondaparinux. In dieser Gruppe wurden 49 % der Patienten verzögert (>24 h) operiert.

Diese theoretischen Überlegungen können auch mit klinischen Daten untermauert werden. Aktuelle Untersuchungen hinsichtlich Blutungskomplikationen bei operativer Versorgung proximaler Femurfrakturen unter ATDT zeichnen hier ein einheitliches Bild. Eine Fall-Kontroll-Studie von Tran et al. an insgesamt 520 Patienten konnte zwischen Patienten ohne ATDT, mit VKA oder mit DOAK keinen Unterschied hinsichtlich der Menge des geschätzten Blutverlustes und der erforderlichen Bluttransfusionen zeigen [27]. Eine retrospektive Studie von Rutenberg et al. an 796 Patienten zeigte ebenso keinen Unterschied in der Menge der benötigten Bluttransfusionen zwischen diesen 3 Gruppen, obgleich auch in dieser Studie Patienten mit ATDT deutlich später operativ versorgt wurden als Patienten ohne ATDT [9]. Eine aktuelle Arbeit der Kollegen Lott et al. zeigte keinen Unterschied bei Patienten unter DOAKs in der Blutungsmenge, der Anzahl der erforderlichen Bluttransfusionen und der Dauer des operativen Eingriffs hinsichtlich eines frühen vs. späten Operationszeitpunktes [17].

Unter Einnahme von Vitamin-K-Antagonisten kam es in der vorgelegten Auswertung zu einer signifikanten Erhöhung des relativen Risikos für das Auftreten von kardiovaskulären Komplikationen, Hämatomen/Nachblutungen sowie von allgemeinen postoperativen Komplikationen. Für das Auftreten „spezifischer postoperativer Komplikationen“ hingegen fand sich hingegen bei keiner Substanzklasse eine signifikante Erhöhung des relativen Risikos. Mattisson et al. untersuchten 99 Patienten, die Warfarin einnahmen und eine proximale Femurfraktur erlitten hatten, und stellten sie 99 Kontrollpatienten mit proximaler Femurfraktur ohne Einnahme eines Antikoagulans (Kontrollgruppe) gegenüber [18]. Auch sie fanden keinen signifikanten Unterschied in Bezug auf Komplikationen. Auch die Mortalität (Einjahres-Follow-up) war im Vergleich zur Kontrollgruppe nicht erhöht. Überraschenderweise fanden sie sogar eine niedrigere Transfusionsrate in der Warfaringruppe. Auch hier sei nochmals auf die eingeschränkte Vergleichbarkeit von Warfarin vs. Phenprocoumon verwiesen.

Die vorgelegte Auswertung zeigt, dass ein Großteil der verzögert operierten Patienten früher operativ versorgt werden könnte und somit die Letalität insgesamt gesenkt werden könnte. Eine aktuelle Veröffentlichung von Bonnaire et al. fasst das Management bei bestehender Antikoagulation und hüftgelenknahen Frakturen zusammen; die Arbeit unterstreicht, dass eine zeitnahe Versorgung im Zusammenhang mit Antikoagulation und hüftnaher Fraktur zum einen essenziell und zum anderen durchaus praktisch möglich ist [4].

### Limitationen

Bei den analysierten Daten handelt es sich um Daten der externen, stationären Qualitätssicherung. Der Vorteil dieser Datenform ist eine hohe Fallzahl (24.786) mit hoher Power. Auf der anderen Seite werden diese Daten zwangsweise erhoben und durch Personal der operierenden Fachabteilung eingegeben, sodass Eingabefehler (bewusst oder unbewusst) nicht auszuschließen sind. Im Falle von Auffälligkeiten ist mit Mehrarbeit/„Sanktionen“ infolge des strukturierten Dialogs zu rechnen. Weiterhin können bei einem derartigen Design keine direkten Kausalzusammenhänge hergestellt werden. Ziel der Auswertung war es, Potenziale für eine Verkürzung der präoperativen Verweildauer aufzuzeigen. Dass die Mortalität durch entsprechende Maßnahmen (Gabe von Antidota etc.) tatsächlich gesenkt werden kann, muss durch entsprechende Studien gezeigt werden. Darüber hinaus fand die Datenerhebung/Analyse nicht sektorenübergreifend statt. Somit können nur die Komplikationen, Todesfälle etc. eines Patienten während des stationären Aufenthaltes erfasst werden. Tritt eine Komplikation, ein Todesfall etc. poststationär bzw. außerhalb der operierenden Fachabteilung auf, wird dies durch die Daten der externen, stationären Qualitätssicherung nicht erfasst. Limitationen ergeben sich auch durch das Erhebungsinstrument. Es wird nach „Art der Medikation“ gefragt. Dabei wird nicht erfasst, wann die letzte Einnahme des Antikoagulans erfolgte. Insbesondere im Hinblick auf die DOAKs wäre diese Angabe relevant.

## Fazit für die Praxis

- Die Einnahme von Antikoagulanzien ist der Hauptgrund für eine verlängerte präoperative Verweildauer.
- Von den Patienten, die verzögert operiert werden (>24 h), nehmen 46,9 % eine ATDT ein.
- Ein Großteil der Patienten könnte durch die Verwendung von Antidota innerhalb von 24 h versorgt werden, und somit könnte die Sterberate vermutlich signifikant gesenkt werden.
- Die Einnahme einer ATDT hat auf thrombotische Komplikationen und Lungenembolie keinen signifikanten Einfluss.
- Unter Einnahme von Vitamin-K-Antagonisten und „anderen Thrombozytenaggregationshemmern“ treten signifikant mehr Hämatome und Nachblutungen auf, nicht aber bei Einnahme von ASS, direkten Thrombininhibitoren und „sonstigen“ Antikoagulanzien.
- Die Ergebnisse müssen vor dem Hintergrund von EQS-Daten bewertet werden.
- Die Etablierung eines Gerinnungsmanagements in Form von SOP in den Kliniken ist zu fordern.

## Footnotes

1 *Allgemeine behandlungsbedürftige postoperative Komplikation(en)*: Pneumonie, kardiovaskuläre Komplikation(en), tiefe Bein‑/Beckenvenenthrombose, Lungenembolie, Sonstige.

2 *Behandlungsbedürftige intra-/postoperative chirurgische Komplikation(en)*: Implantatfehllage, Implantatdislokation, Wundhämatom/Nachblutung, Gefäßläsion, Nervenschaden, Fraktur, Sonstige.

## References

[1] Aqua-Institut (2014) Bundesauswertung proximaler Femurfrakturen. https://www.sqg.de/downloads/Bundesauswertungen/2014/bu_Gesamt_17N1-HUEFT-FRAK_2014.pdf. Zugegriffen: 1. Okt. 2019

[2] Arbeitsgemeinschaft Der Wissenschaftlichen Medizinischen Fachgesellschaften (2015, Herausgegeben am 10.04.2014, zuletzt bearbeitet am 9.10.2015, Gültig bis 9. Okt. 2020) S2e Leitlinie „Schenkelhalsfraktur des Erwachsenen“

[3] Biberthaler P (2018). Versorgungsforschung. Unfallchirurg.

[4] Bonnaire F, Bula P, Schellong S (2019). Management vorbestehender Antikoagulation zur zeitgerechten Versorgung von hüftnahen Frakturen. Unfallchirurg.

[5] Buecking B, Eschbach D, Bliemel C (2014). Effectiveness of vitamin K in anticoagulation reversal for hip fracture surgery—a prospective observational study. Thromb Res.

[6] Connolly SJ, Milling TJ, Eikelboom JW (2016). Andexanet alfa for acute major bleeding associated with factor Xa inhibitors. N Engl J Med.

[7] Ema (2019). First antidote for reversal of anticoagulation with factor Xa inhibitors apixaban and rivaroxaban.

[8] Fachgesellschaften ADWM (2015, Herausgegeben am 10.04.2015, Zuletzt bearbeitet am 10.02.2015, Gültig bis 9. Febr. 2019) S2e Leitlinie „Pertrochantäre Femurfraktur“.

[9] Frenkel Rutenberg T, Velkes S, Vitenberg M (2018). Morbidity and mortality after fragility hip fracture surgery in patients receiving vitamin K antagonists and direct oral anticoagulants. Thromb Res.

[10] G‑Ba (2019) Beschluss des Gemeinsamen Bundesausschusses über eine Richtlinie zur Versorgung der hüftgelenknahen Femurfraktur. https://www.g-ba.de/downloads/39-261-4069/2019-11-22_QSFFx-RL_Erstfassung.pdf. Zugegriffen: 22. Feb. 2020

[11] Grottke O, Akman N, Conley PB (2017). Abstract 20205: the impact of Andexanet alfa in a porcine polytrauma model under apixaban anticoagulation: investigation of hemostatic safety and efficacy. Circulation.

[12] Grottke O, Honickel M, Van Ryn J (2015). Idarucizumab, a specific dabigatran reversal agent, reduces blood loss in a porcine model of trauma with dabigatran anticoagulation. J Am Coll Cardiol.

[13] Heo YA (2018). Andexanet alfa in the treatment of acute major bleeding related to apixaban and rivaroxaban: a profile of its use in the USA. Drugs Ther Perspect.

[14] Iqtig (2016). Hüftgelenknahe Femurfraktur mit osteosynthetischer Versorgung – Indikatoren.

[15] Iqtig (2019) IQTIG Qualitätsbericht für das Erfassungsjahr 2018. https://iqtig.org/qs-berichte/strukturierterqualitaetsbericht/#category42. Zugegriffen: 25. Feb. 2020

[16] Klestil T, Roder C, Stotter C (2017). Immediate versus delayed surgery for hip fractures in the elderly patients: a protocol for a systematic review and meta-analysis. Syst Rev.

[17] Lott A, Haglin J, Belayneh R (2019). Surgical delay is not warranted for patients with hip fractures receiving non-warfarin anticoagulants. Orthopedics..

[18] Mattisson L, Lapidus LJ, Enocson A (2018). Is fast reversal and early surgery (within 24 h) in patients on warfarin medication with trochanteric hip fractures safe? A case-control study. BMC Musculoskelet Disord.

[19] Mo Y, Yam FK (2015). Recent advances in the development of specific antidotes for target-specific oral anticoagulants. Pharmacotherapy.

[20] Müller-Mai CM, Schulze Raestrup US, Kostuj T (2015). Einjahresverläufe nach proximalen Femurfrakturen. Unfallchirurg.

[21] Ng R, Shabani-Rad MT (2019). Results of Octaplex for reversal of warfarin anticoagulation in patients with hip fracture. Can J Surg.

[22] Patanwala AE, Acquisto NM, Erstad BL (2011). Prothrombin complex concentrate for critical bleeding. Ann Pharmacother.

[23] Pollack CV, Reilly PA, Eikelboom J (2015). Idarucizumab for dabigatran reversal. N Engl J Med.

[24] Schwabe U, Paffrath D, Ludwig W (2018). Arzneiverordnungs-Report 2018.

[25] Siegal DM, Curnutte JT, Connolly SJ (2015). Andexanet alfa for the reversal of factor Xa inhibitor activity. N Engl J Med.

[26] Steffel J, Verhamme P, Potpara TS (2018). The 2018 European Heart Rhythm Association Practical Guide on the use of non-vitamin K antagonist oral anticoagulants in patients with atrial fibrillation. Eur Heart J.

[27] Tran T, Delluc A, De Wit C (2015). The impact of oral anticoagulation on time to surgery in patients hospitalized with hip fracture. Thromb Res.

